# Increased innate immune responses in adolescents with obesity and its relation to subclinical cardiovascular measures: An exploratory study

**DOI:** 10.1016/j.isci.2024.109762

**Published:** 2024-04-23

**Authors:** Siroon Bekkering, Christoph Saner, Boris Novakovic, Toby Mansell, Danielle K. Longmore, Zoe McCallum, Anne-Louise Ponsonby, Markus Juonala, Mihai G. Netea, Matthew A. Sabin, Richard Saffery, Niels P. Riksen, David P. Burgner

**Affiliations:** 1 Department of Internal Medicine, Radboud University Medical Centre, Nijmegen 6525 GA, the Netherlands; 2 Murdoch Children’s Research Institute, The Royal Children’s Hospital, Parkville, VIC 3052, Australia; 3 Division of Pediatric Endocrinology, Diabetology and Metabolism, Department of Pediatrics, University Children`s Hospital Bern, Inselspital, Bern 3010, Switzerland; 4 Department of Biomedical Research, University of Bern, 3010 Bern, Switzerland; 5 Department of Endocrinology, The Royal Children`s Hospital, Parkville, VIC 3052, Australia; 6 Department of Paediatrics, The University of Melbourne, Parkville, VIC 3052, Australia; 7 Department of Neurodevelopment and Disability, Royal Children’s Hospital, Parkville, VIC 3052, Australia; 8 Florey Institute of Neuroscience and Mental Health, University of Melbourne, Parkville, VIC 3052, Australia; 9 Department of Medicine, University of Turku, 20500 Turku, Finland; 10 Division of Medicine, Turku University Hospital, 20500 Turku, Finland; 11 Department of Immunology and Metabolism, Life and Medical Sciences Institute, University of Bonn, 53115 Bonn, Germany; 12 Department of Pediatrics, Monash University, Clayton, VIC 3168, Australia

**Keywords:** Health sciences, Immunology, Pathophysiology, Disease, Risk factor

## Abstract

Cardiometabolic risk accrues across the life course and childhood and adolescence are key periods for effective prevention. Obesity is associated with inflammation in adults, but pediatric data are scarce. In a cross-sectional and longitudinal study, we investigated immune cell composition and activation in 31 adolescents with obesity (41.9% male, BMIz>2.5, 14.4 years) and 22 controls with healthy weight (45.1% male, −1.5<BMIz<1.5, 14 years). In those with obesity, we assessed the impact of weight change and correlations between immune profiles and subclinical cardiovascular phenotypes at a 5-year follow-up. Compared to controls, those with obesity had increased monocyte activation and cytokine production upon stimulation. Monocyte transcriptomics demonstrated upregulated inflammatory pathways and downregulated antiviral responses. Weight change was not associated with changes in inflammation. Baseline inflammation correlated with cardiovascular measures at follow-up. Children with obesity have increased inflammation, which associates with worse subsequent subclinical cardiovascular measures. Adjunctive anti-inflammatory interventions may be needed to reverse adverse subclinical cardiovascular phenotypes.

## Highlights

• 31 children with obesity and 22 healthy controls underwent in-depth immune phenotyping
• Those with obesity have increased systemic and cellular inflammation
• There are no strong effects of change in BMI on inflammation
• Childhood inflammation associates with subclinical cardiovascular measures in adolescence

## Graphical abstract

**Figure:**
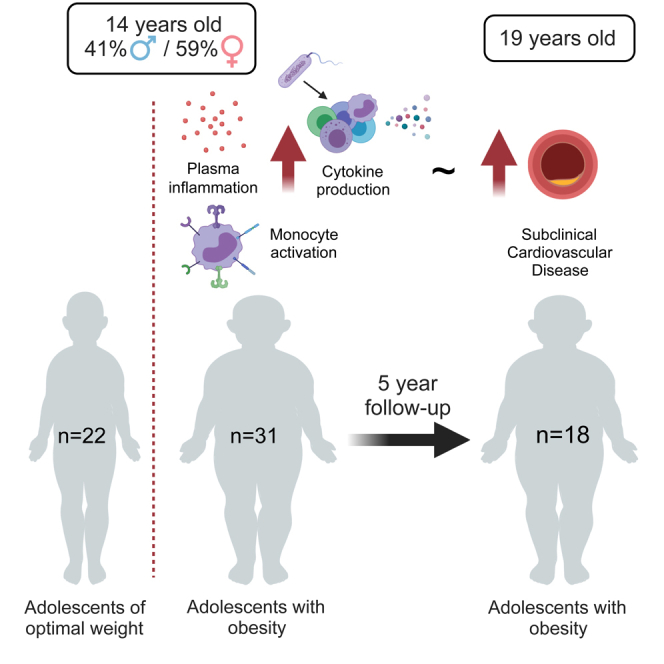

## Introduction

Atherosclerotic cardiovascular disease (ASCVD) is the leading cause of mortality, responsible for one-third of all annual deaths worldwide.^1^ Obesity is a major modifiable risk factor for ASCVD^2^^,^^3^ and the global prevalence of obesity continues to increase. Importantly, the prevalence of obesity in children is also increasing.^4^ Children with obesity have a 5-fold higher chance of living with obesity in adulthood.^5^^,^^6^ Childhood obesity is also associated with markedly increased risk for metabolic disease and impaired vascular health in adulthood.^7^ If children with overweight or obesity regain healthy body mass index (BMI) as adults, cardiometabolic risk (i.e., risk of type 2 diabetes, hypertension, adverse plasma lipid profiles, and carotid artery atherosclerosis) largely attenuates to baseline,^5^ highlighting the importance of interventions early in life. On the other hand, sustained weight loss, either through bariatric surgery or due to lifestyle changes, has shown no benefit on the development of coronary artery disease.^8^ Interestingly, recent experimental studies have shown immune reprogramming by transient obesity that persists despite returning to normal weight,^9^ which could potentially contribute to later ASCVD.

Inflammation is a central pathogenic mechanism in the development of ASCVD and predicts CVD events. Randomized clinical trials in individuals with previous CVD events provide proof that reducing inflammation is effective as secondary prevention, independent of changes in lipid profile.^10^^,^^11^ Obesity is a pro-inflammatory state and, in adults, chronic inflammation mediates much of the adverse cardiometabolic consequences of obesity.^12^ There are much less data available for children and adolescents. Among Australian Aboriginal children with obesity, high-sensitivity c-reactive protein (hsCRP) is associated with adverse cardiovascular (CV) measures.^13^ However, hsCRP has no clear causal role^14^ and is a relatively poor biomarker of chronic inflammation, especially in childhood.^15^ Chronic inflammation (i.e., increased hsCRP and circulating protein biomarkers of inflammation) is reported among children and adolescents with obesity,^16^ but it is unknown if underlying cellular immune phenotypes differ, whether inflammatory responses alter with change in weight status and how this relates to cardiometabolic risk phenotypes. Functional assessment of inflammation (i.e., cellular markers of inflammation, cytokine production capacity and transcriptomics) may further elucidate underlying mechanisms and identify additional targets to reduce the adverse cardiometabolic effects of obesity.^17^

To address these knowledge gaps, we aimed to assess the innate immune phenotype of children and adolescents with obesity using a systems biology approach, including measurement of plasma protein biomarkers of inflammation, flow cytometric assessment of cellular immune phenotypes, functional assays of innate immune cytokine production capacity, and transcriptomics of isolated immune cells. We evaluated immune phenotypes of children and adolescents with obesity and compared findings with controls of similar age and sex with BMI in the healthy range. Those with obesity were subsequently followed for a mean of 5.5 years. We assessed associations between BMI change with longitudinal changes in these innate immune parameters and associations between immune parameters on both timepoints and non-invasive CV measures at follow-up.

## Results

### Study participants at baseline *–* Those with obesity vs. controls

**Figure 1:**
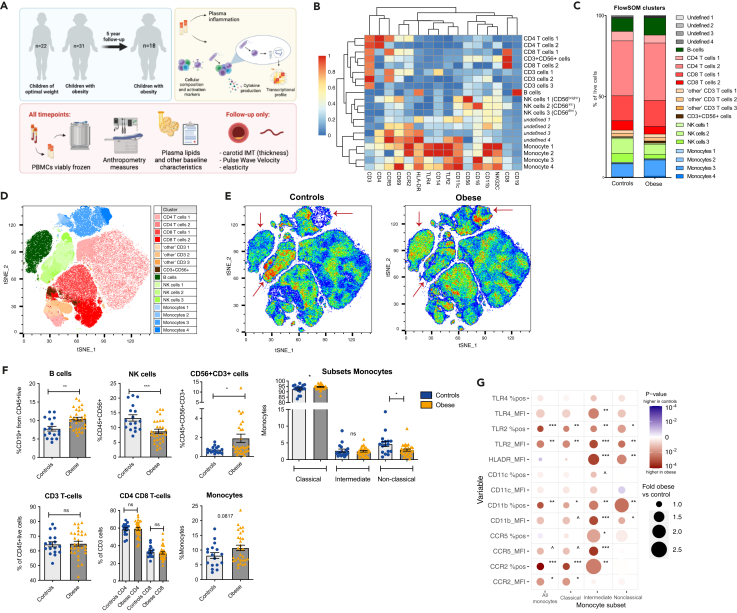
Study setup and flow cytometric analysis of circulating immune cells of controls (*n* = 22) and those with obesity (*n* = 31) (A) Graphical overview of study setup. (B) FlowSOM un-hierarchical clustering of samples to identify cellular subtypes. (C) Cellular subtypes per study: controls or obese. (D) tSNE visualization of cellular subtypes. (E) tSNE visualization per group. Three marked different cellular subtypes can be observed as pointed out with arrows: Monocytes, NK cells and B cells. (F) Manual gating confirming the cellular subtypes in children with obesity and children of optimal weight. Those with obesity are indicated in yellow and controls in blue. Figures show all individual data plus bars showing the mean and Standard Error of the Mean (SEM). (G) Monocyte activation markers (% positive and median fluorescent intensity) of all monocytes or on monocyte subtypes. The size of the circles indicates the difference of obese over controls (the larger the circle, the higher in those with obesity) and the color indicates the *p*-value.

We selected 31 children and adolescents with obesity from the original COBRA cohort and 22 controls, with a comparable age and sex distribution (Figure 1A; Table 1). Thirteen (41.9%) of the obese participants were male and the median age was 14.4 years. For controls, 10 (45.1%) were male, and the median age was 14.0 years. All participants were postpubertal. Mean BMI in those with obesity was 37.3 kg/m^2^±5.9, corresponding to an age- and sex-adjusted mean BMIz of 2.5 ± 0.3. Controls had a mean BMI of 20.8 ± 3.2, corresponding to a mean BMIz of 0.3 ± 0.9. None of the children had inflammatory co-morbidities or were on anti-inflammatory medication. Total plasma cholesterol and triglycerides were comparable between groups. LDL- and HDL-cholesterol were within normal range but slightly higher (2.5 mmol/L vs. 2.1 mmol/L) and lower (1.1 mmol/L vs. 1.3 mmol/L) respectively in those with obesity compared to controls. Non-HDL-c was similar between groups.

**Table 1:** Baseline characteristics of those with obesity vs. those of healthy weight

	Subjects with obesity (*n* = 31)	Controls (*n* = 22)	*p*-value
Age, median [range]	14.4 [10.1–17.5]	14.0 [10.0–17.0]	ns
Sex, n(%male)	13(41.9)	14(45.1)	ns
Height(cm), mean ± SD	166 ± 9	166 ± 12	ns
Weight(kg), mean ± SD	104 ± 21	58 ± 11	<0.0001
BMI(kg/m^2^), mean ± SD	37.3 ± 5.9	20.8 ± 3.2	<0.0001
BMI *Z* score	2.5 ± 0.3	0.3 ± 0.9	<0.0001
BMI>95^th^ percentile (%)	139.4 ± 20.2	81.1 ± 13.4	<0.0001
Medication (n) Metformin Lantus (insulin) Glibenclamide	1 2 1	NA	
TC (mmol/L)	4.15 ± 0.63	4.01 ± 0.77	ns
LDL-C (mmol/L)	2.46 ± 0.56	2.08 ± 0.63	0.03
HDL-C (mmol/L)	1.07 ± 0.20	1.27 ± 0.33	0.01
TG (mmol/L)	1.36 ± 0.77	1.45 ± 0.74	ns
Non-HDL-C (mmol/L)	3.07 ± 0.56	2.74 ± 0.70	ns
Plasma IL-6 (pg/mL)	1.79 [0.16–10.2]	0.50 [0.21–1.74]	<0.0001
Plasma hsCRP (mg/dL)	2.10 [0.16–12.4]	0.24 [0.16–2.63]	<0.0001

Values are n(%), mean ± SD for normally distributed data or median [range] for non-normally distributed data. BMI, body mass index; TC, total cholesterol; LDL-C, low-density lipoprotein cholesterol; HDL-C, high-density lipoprotein cholesterol; TG, triglycerides; IL, interleukin; CRP, C-reactive protein; SD, standard deviation.

#### Children and adolescents with obesity have higher systemic inflammation

To investigate systemic inflammation, we quantified plasma concentrations of IL-6 and hsCRP. Individuals with obesity showed higher plasma levels of IL-6 (median 1.79 vs. 0.50 pg/mL in controls) and hsCRP (median 2.10 vs. 0.24 mg/dL in controls) (Table 1). There were no sex differences in systemic inflammation. (Figure S1B).

#### Children and adolescents with obesity have marked differences in PBMC composition and increased activation of circulating monocytes

To investigate whether obesity was associated with changes in the immune phenotype in circulating immune cells, we assessed the composition and activation state of PBMCs in both groups using high dimensional flow cytometry. The 20 meta-clusters identified using FlowSOM were further annotated manually and analyzed for differences between groups. Based on marker expression, clusters were classified as B cells (9.1% of live CD45^+^ cells), CD4^+^ T cells (2 clusters, a total of 41.9%), CD8^+^ T cells (2 clusters, a total of 20.7%), double-negative (CD4^−^CD8^−^) CD3^+^ T cells (3 clusters, a total of 4.2%), NK cells (3 clusters, a total of 10.7%), CD3^+^CD56^+^ cells (1%) and monocytes (4 clusters, a total of 11%), leaving 4 clusters undefined (a total of 1.5%) (Figure 1B). The frequencies of the clusters for each study population is shown in Figure 1C. The nonlinear dimensionality reduction technique t-distributed stochastic neighbor embedding (tSNE) further visualized these high dimensional data (Figures 1D and S1D). The cells are color-coded by their respective FlowSOM cluster (Figure 1D), and the color highlighting was confirmed using manual gating (Figure S1E). The respective tSNEs from those with obesity and controls are shown in Figure 1E, with differences in the monocyte, NK and B cell clusters highlighted. Manual gating confirmed a significantly higher number of B-cells in those with obesity, a reduced number of NK cells, and an increase in the percentage of CD3^+^CD56^+^ cells. The percentage of T cells and CD4 and CD8 T cell subsets did not differ between groups (Figure 1F), but T-cells in children with obesity showed a lower expression of CCR5 (Figure S1F). B-cells from children with obesity have higher expression of HLA-DR (Figure S1F). The number of monocytes was only modestly higher in those with obesity, but monocyte subset analysis based on CD14 and CD16 expression showed a marked higher percentage of classical monocytes (CD14++ CD16dim) and lower percentage of non-classical monocytes (CD14dimCD16++) in subjects with obesity (Figure 1F). There were no sex differences in the proportions of circulating immune cells (Figure S1G).

The activation state of the monocytes was assessed by the percentage of cells stained positive for a marker as well as the median expression of activation markers (MFI) on the positive cells. We assessed human leukocyte antigen-DR (HLA-DR), cluster of differentiation (CD)11b, CD11c, chemokine receptor type 2 (CCR2), CCR5, toll-like receptor (TLR)4 and TLR2. Monocytes from those with obesity had significantly higher expression of all activation markers (both percentage and MFI) compared to controls (Figure 1G; Table S1). Analyses of the activation markers on the three monocyte subsets showed more marked differences between groups (Figure 1G). There were some sex differences in the expression of monocyte activation markers: in children with obesity, expression (MFI) of CCR2, CD11b, CD11c, TLR2 and TLR4 were higher in females than males indicating that monocytes in female children with obesity have a more pro-inflammatory phenotype (Figure S1H; Table S2).

#### Increased cytokine production capacity in monocytes from those with obesity

**Figure 2:**
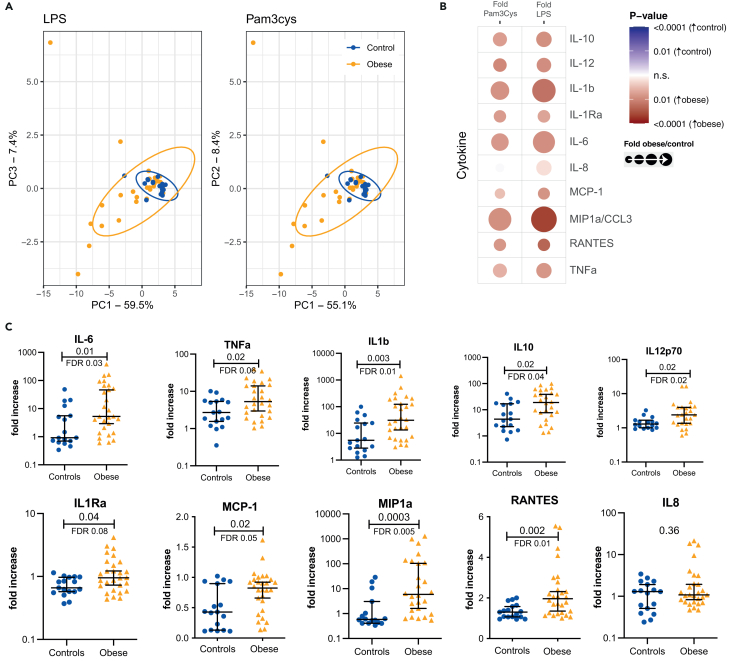
Cytokine production capacity upon *ex vivo* stimulation with LPS (TLR4) or Pam3Cys (TLR2) or medium as negative control Cytokines were measured in the supernatant after 24 h and assessed using Bio-Plex 27-cytokine arrays. (A) PCA plot indicating all cytokines per participant for LPS and Pam3Cys stimulation. Controls cluster together whereas obese separate and are more variable. (B) Manual assessment of cytokine production capacity. Circles indicate the fold of measures in children with obesity over children of optimal weight. Colors indicate the *p*-value. (C) Individual values of innate cytokine production capacity (stimulated over baseline) for LPS stimulated PBMCs. Those with obesity are indicated in yellow and controls in blue. Figures show all individual data plus mean with interquartile range.

Next, we assessed cytokine and chemokine production capacity of PBMCs. We measured cytokines after *ex vivo* stimulation with either culture medium as negative control or LPS and Pam3Cys, ligands for TLR4 and TLR2 respectively. Principal Component Analysis of all 27 proteins showed marked differences between the cytokine production capacity (stimulated over baseline production, i.e., fold increase) of children with obesity and controls (Figure 2A), with similar changes observed with each stimulus. We then focused on a selection of 10 innate cytokines and chemokines known to be important in CVD, as depicted in the heatmap (Figure 2B). The cytokine production capacity of children with obesity was higher for TNF, IL-1β, IL-6, IL-10, IL-1Ra, IL-12p70, MCP-1, RANTES, and MIP1α, but not IL-8 (Figures 2B and 2C; Table S3; Figure S2A), indicating an overall hyperresponsive state compared to controls. There were no clear sex differences in cytokine production capacity, although some cytokines were higher in females with obesity compared to males (for IL-1β, IL-6, TNF), in line with findings on activation markers by flow cytometry (Figure S2B; Table S4).

#### Unstimulated and stimulated monocytes from those with obesity have a distinct transcriptional profile

**Figure 3:**
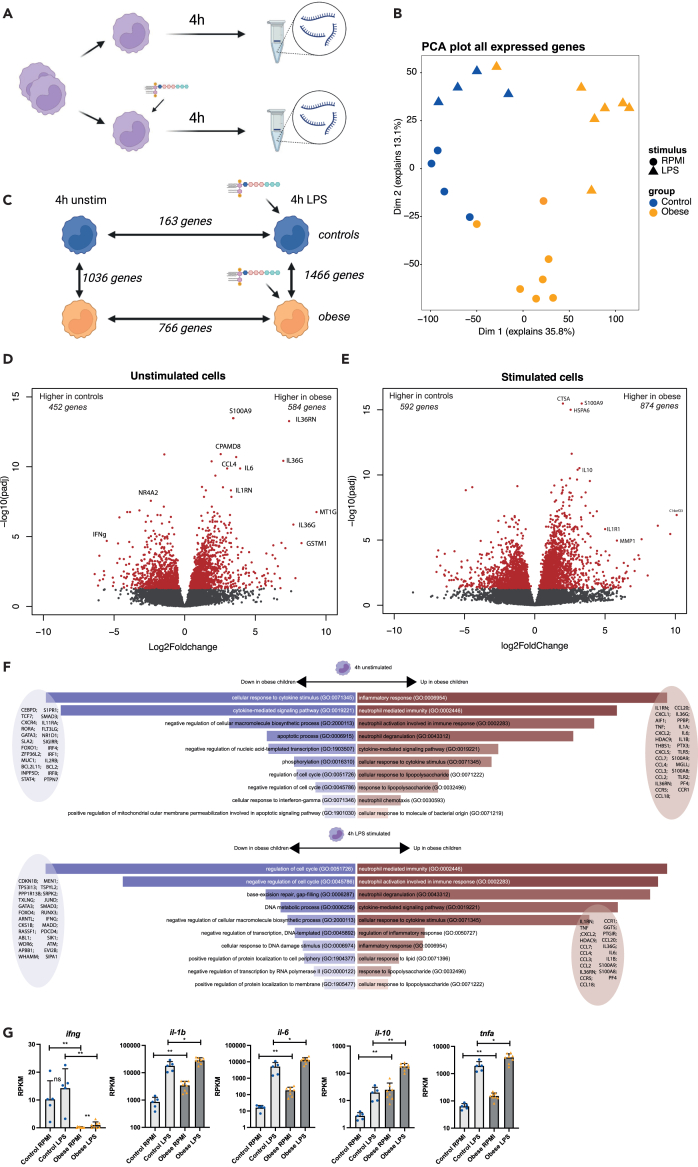
Transcriptomic analysis of isolated monocytes from children with obesity and children of optimal weight (A) Experimental set-up. Monocytes were isolated and stimulated for 4 h using medium (unstimulated controls) or LPS. After 4 h, RNA was isolated and used for RNA-seq analysis. (B) PCA plot showing all expressed genes (RPKM>5) for each individual. Controls and those with obesity clearly separate as well as RPMI or LPS stimulated cells. (C) Differentially expressed genes, graphical overview. (D) Volcano plot showing differences between controls and those with obesity in unstimulated monocytes. Red color indicates significantly upregulated (right) or downregulated (left) genes in children with obesity. (E) Volcano plot showing differences between controls and obese in LPS stimulated monocytes. Red color indicates significantly upregulated (right) or downregulated (left) genes in children with obesity. (F) GO pathway analysis on DEGs. Top panel shows DEG pathways in unstimulated monocytes. Lower panel shows DEG pathways in LPS stimulated monocytes. Some genes in pathways are shown. (G) RPKM values of *ifng*, *il-6*, *tnf*, *il-1β* and *il-10* in unstimulated and stimulated monocytes from those with obesity and controls. Those with obesity are indicated in yellow and controls in blue. Figures show all individual data plus bars with SEM.

To further delineate the inflammatory profile of circulating monocytes, we performed RNA sequencing analysis (RNA-seq) on isolated monocytes stimulated for 4 h with medium (unstimulated) or LPS (stimulated, Figure 3A).

Based on all expressed genes (RPKM>5), monocytes from subjects with obesity clearly separated from controls in PCA (Figure 3B) in unstimulated conditions and following LPS stimulation. Compared to unstimulated, 766 genes were upregulated in those with obesity following LPS stimulation, whereas in controls, only 163 genes were significantly upregulated (Figure 3C). Comparison of the 4-h unstimulated monocytes from obese with 4 h unstimulated monocytes from controls, showed 1036 significantly different genes (P-adj<0.05, fold change>2), of which 584 were upregulated and 452 downregulated (Figure 3D). Comparison of 4-h LPS stimulated genes between groups showed in 1466 significantly DEGs, of which 874 were upregulated and 592 downregulated (Figure 3E). Comparing all gene sets together, 2166 genes were differentially expressed between groups (p-adj<0.05, Table S5).

In 4 h unstimulated cells, IFNγ was the gene with the most significantly lower expression in adolescents with obesity compared to controls (Figure 3D). In line with this, significantly downregulated GO pathways of DEGs included both “cellular response to interferon-gamma” and “T cell activation” (Figure 3F; Table S6). Significantly upregulated GO pathways in unstimulated monocytes from obese individuals include neutrophil pathways as well as general “inflammatory response” and “response to lipopolysaccharide” pathways (Figure 3F; Table S6). Upon LPS stimulation, downregulated GO pathways in those with obesity include cell cycle regulation as well as apoptosis (Figure 3F; Table S7 for DEGs). More pronounced were upregulated pathways upon LPS stimulation, which again included multiple neutrophil activation pathways, “cytokine mediated signaling”, “cellular response to LPS” and “chemotaxis” (Table S7). Significantly upregulated genes in those with obesity following LPS stimulation included pro-inflammatory (*tnfa*, *il1b* and *il6*) and anti-inflammatory (*il-10*) cytokines (Figure 3G for RPKM values; Table S5 for all differentially expressed genes), confirming the findings at the protein level. Overall, monocytes from adolescents with obesity have a transcriptionally distinct and pro-inflammatory phenotype with increased transcriptional responses upon LPS stimulation compared to control children, whilst having a decreased IFNg signature at the same time.

#### Inflammatory changes over time and adverse cardiovascular measures at follow-up

##### Comparisons between those with obesity at baseline and at follow-up

18 participants (7 male) of the initial 31 had subsequent follow-up assessment and biosamples as part of the COBRA-CVR sub-study (Figure 1A). Baseline characteristics are shown in Table 2. The mean follow-up time was 5.5 [range 3.1–8.4] years and the adolescents had a mean age of 20.3 [range 15.1–24.3] years at follow-up. Subclinical cardiovascular measures were assessed only at follow-up. Height, weight and BMI changed significantly between initial assessment and COBRA-CVR follow-up (Table 2). Total cholesterol, LDL-cholesterol and triglycerides significantly decreased for all participants, independently of BMI change. HbA1c showed significant correlation with change in BMIz (R = 0.66, *p* = 0.02). None of the other baseline characteristics correlated with change in BMIz over time. Determined by change in BMIz, eight participants (44%) decreased, six (33%) increased the severity of their obesity severity, and four (22%) remained approximately the same. One participant reached a BMI in the healthy range. Subsequent analyses investigated the relationship between change in BMIz between time points and change in immune parameters rather than the mean/median values of the total group.

**Table 2:** Baseline characteristics of children with obesity visit 1 vs. visit 2

	Adolescents with obesity baseline (*n* = 18)	Adolescents with obesity follow-up (*n* = 18)	*p*-value
Age, median [range]	14.0 [10.1–16.6]	20.3 [15.1–24.3]	
Sex, n(%male)	7 (38.9)	NA	NA
Height(cm), mean ± SD	167 ± 9	172 ± 10	0.0008
Weight(kg), mean ± SD	99 ± 19	117 ± 25.7	0.0009
BMI(kg/m^2^), mean ± SD	35.4 ± 5.7	39.5 ± 7.8	0.006
BMI *Z* score	2.4 ± 0.3	2.28 ± 0.4	ns
BMI>95^th^ percentile (%)	133.7 ± 20.8	133.7 ± 26.2	ns
Waist circumference (cm)	111 ± 12	113 ± 14	ns
TC (mmol/L)	4.2 ± 0.7	3.3 ± 0.6	<0.0001
LDL-C (mmol/L)	1.5 ± 0.2	1.1 ± 0.3	<0.0001
HDL-C (mmol/L)	1.1 ± 0.2	1.2 ± 0.2	ns
TG (mmol/L)	1.6 ± 0.9	1.2 ± 0.4	0.01
SBP	120.2 ± 14.4	129.9 ± 10.1	0.02
DBP	70.7 ± 9.2	71.8 ± 6.7	ns
Body Fat (%)	43.3 ± 7.2	42.2 ± 9.3	ns
Truncal Fat (%)	37.6 ± 8.1	40.4 ± 8.1	ns
HbA1c	5.35 ± 0.57	5.22 ± 0.64	ns
Total white blood cells (x10ˆ9/mL)	8.4 ± 2.6	7.2 ± 1.2	ns
Plasma IL-6 (pg/mL)	2.0 ± 1.1	2.3 ± 1.8	ns
Plasma CRP (mg/dL)	2.6 ± 2.1	4.1 ± 3.9	ns

Values are n(%) or mean ± SD. BMI, body mass index; TC, total cholesterol; LDL-C, low-density lipoprotein cholesterol; HDL-C, high-density lipoprotein cholesterol; TG, triglycerides; SBP, systolic blood pressure; DBP, diastolic blood pressure; HbA1c, hemoglobin A1c; SD, standard deviation.

##### BMI change over time did not affect monocyte activation

**Figure 4:**
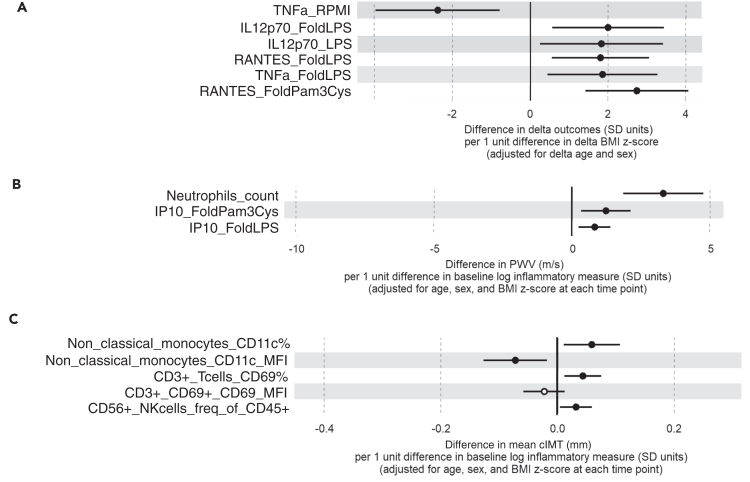
Inflammation at baseline associates with worsened subclinical cardiovascular measures at follow-up (A) Change in BMIz associates with some change in inflammatory measures. Corrected for change in age and sex. (B) Increase in Pulse Wave Velocity (m/s) per unit difference in log transformed baseline inflammatory measures. (C) Difference in cIMT (mm) per unit difference in log transformed baseline inflammatory measures.

We studied monocyte subsets and activation markers (by flow cytometry) and cytokine production capacity upon *ex vivo* stimulation at both timepoints. Change in BMIz showed a correlation with change in inflammation for some measures, but sensitivity analysis showed that these were driven by the one participant who obtained a weight in the healthy range (data not shown). Excluding this participant, three correlations between change in BMIz and inflammatory measures remained, such as a 2-fold increase in the production of RANTES upon both LPS and Pam3Cys stimulation with an increase of 1 BMIz and similarly for TNF production upon LPS stimulation (Figure 4A; Table S8). Overall, most markers did not change significantly.

#### Inflammation at baseline associates with subsequent adverse subclinical cardiovascular measures in those with obesity

All participants in COBRA-CVR had subclinical cardiovascular measures assessed at follow-up (PWV, carotid IMT (cIMT), carotid elasticity and blood pressure). We performed regression analysis with a model correcting for age, sex and BMIz. None of the inflammatory parameters at follow-up were cross-sectionally associated with any of the CV measures at follow-up (data not shown). On the other hand, several measures of inflammation at baseline were associated with CV measures at follow-up independent of BMIz (Table S9). For example, higher neutrophil numbers at baseline associated with higher PWV at follow-up (Figure 4B; Table S9). In addition, increased cytokine production of interferon inducible protein 10 (IP10) following LPS and Pam3Cys stimulation, was associated with higher PWV at follow-up (Figure 4B; Table S9). Some flow cytometric markers of inflammation at baseline were associated with cIMT at follow-up (Figure 4C). The percentage of CD11c expressing non-classical monocytes at baseline associated with higher cIMT at follow-up (Figure 4C). The number of CD56 NK cells and activated T-cells (CD69 expressing T cells) were also positively associated with higher cIMT at follow-up. The number of CD11b expressing intermediate monocytes at baseline associated with lower cIMT at follow-up (Figure 4C; Table S9). None of the inflammatory measures at baseline were associated with carotid artery elasticity at follow-up (data not shown).

## Discussion

In this study, we report that monocytes from children and adolescents with severe obesity have a pro-inflammatory state compared to controls of the same age with healthy BMI. We assessed inflammation using several methodological approaches and show consistently heightened inflammatory parameters and responses in those with obesity. Compared with controls, those with obesity have increased systemic inflammatory biomarkers; differences in cellular composition and most strikingly, differences in cellular activation markers. Monocytes from those with obesity have an increased innate cytokine production capacity when exposed to TLR ligands. Transcriptomic analysis using RNA sequencing showed marked differences in both unstimulated and stimulated isolated monocytes between adolescents with obesity and controls: those with obesity had a more marked pro-inflammatory phenotype, but decreased anti-viral responses. Secondly, we show that there was no clear correlation between prospective changes in BMI in those with obesity and inflammatory measures. Finally, we found an association between baseline inflammatory markers and subclinical cardiovascular measures at follow-up in those with obesity.

Obesity is a low-grade systemic inflammatory state and a key risk factor for ASCVD, but the underlying mechanisms are incompletely understood. There is considerable evidence that adults with obesity have heightened inflammation.^17^ Earlier intervention is likely to be effective in preventing CVD,^18^ but data on inflammation from children and adolescents with obesity are scarce and less consistent.^17^ Previous studies have shown increased numbers of monocytes in children with obesity^19^^,^^20^ but less consistent differences in monocyte subsets,^20^^,^^21^^,^^22^^,^^23^^,^^24^ with either an increase in non-classical monocytes,^21^ classical monocytes^20^ or both,^22^^,^^24^ which can be explained mainly by methodological differences. Inconsistent data from cytokine production analyses, with some studies showing higher cytokine responses upon stimulation^23^^,^^25^ in children with obesity, whereas others showed a lower response,^21^ may also reflect methodological issues. For example, if one study measures cytokines in the supernatant and the other intracellularly using flow cytometry, comparisons across studies are problematic. A cross-sectional analysis compared gene expression profiles in children with obesity with healthy weight controls and found a distinct monocyte gene expression pattern,^22^ although the control group was considerably younger, again limiting interpretation. To our knowledge, there are no longitudinal data on immune phenotypes and intermediate outcomes for cardiovascular disease in children and adolescents with severe obesity.

In this study, we used a systems biology approach to assess inflammation cross-sectionally in post-pubertal adolescents with healthy BMI versus obesity, and longitudinally in individuals with obesity by assessing a number of parameters. Our findings highlight the complex and nuanced immune phenotype in this population; monocytes from children and adolescents with obesity have a shift in subsets, and are both more pro-inflammatory in response to stimulation with some TLR ligands, but concomitantly show reduced anti-viral responses. These findings are in line with heightened pro-inflammatory innate and inflammatory responses reported from adults with obesity,^26^^,^^27^ who have higher monocyte numbers,^26^^,^^27^ changes in monocyte subsets and increased activation markers on flow cytometry.^28^^,^^29^ The sub-optimal anti-viral response is in keeping with epidemiological data which show increased disease severity and worse outcomes with SARS-COV-2^30^ and influenza^31^ in this population. Here we also show that higher inflammation at baseline among those with obesity is associated with adverse cardiovascular measures at follow-up.

There are several plausible mechanisms contributing to heightened inflammation in adults and children with obesity. These include inflammatory cytokine production by expanded adipose tissue,^32^ chronic activation of the immune system by metabolic endotoxemia,^33^ and the recently described long-term activation of the innate immune system. This latter paradigm, known as innate immune memory, or trained immunity, is characterized by stable epigenetic and metabolic reprogramming of monocytes and macrophages upon stimulation, leading to enhanced responses upon secondary stimulation.^34^ Exposure to endogenous pro-inflammatory stimuli, such as high levels of (oxidized) LDL,^35^ glucose,^36^ a Western diet^37^ or metabolic endotoxemia^38^ have all been shown to induce innate immune reprogramming in murine, human, and *in vitro* models. Innate immune memory results in persistent inflammation even upon removal of the stimulus. Recently, in a murine model of neuroinflammation, a history of previous obesity induced persistent changes in innate immune cells.^9^ In this model, despite unchanged myeloid cell numbers upon weight loss, adipose tissue macrophages and haematopoietic stem cells had sustained pro-inflammatory phenotypes, with epigenetic changes in pro-inflammatory genes.^9^ Our data are suggestive of a trained immunity phenotype (i.e., increased cytokine production capacity and transcriptional changes, which do not change upon weight loss), although further studies confirming epigenetic and metabolic reprogramming are needed. Interestingly, our transcriptomic data also showed signatures of upregulated neutrophil activation pathways and neutrophil numbers at baseline were associated with increased arterial stiffness at follow-up. Future studies should investigate neutrophils in more detail, since recent evidence points toward a role for neutrophils in cardiovascular disease^39^ as well as in trained immunity.^40^

Several therapeutic options are available for individual modifiable CVD risk factors in adults, but lifestyle treatment options for children with obesity have limited sustained success.^41^ In addition, some risk factors for childhood obesity (e.g., socioeconomic status and heritability) are difficult to modify or are fixed. Importantly, if children with obesity achieve BMI within the healthy range by adulthood, the risk for several CVD risk factors (i.e., risk of type 2 diabetes, hypertension, adverse plasma lipid profiles, and carotid artery atherosclerosis) later in life drops significantly.^5^ However, to date, weight loss has not been demonstrated to reduce coronary artery disease rates.^8^ In addition, previous studies have not investigated sustained inflammation following weight loss and its potential contribution to future CVD risk in those with obesity. It may be more meaningful to focus on outcomes, such as metabolic dysfunction or inflammation, rather than BMI or adiposity per se, as the former are likely to underpin the increase in cardiometabolic disease and related risk later in life.^42^ Inflammation is now also recognized as residual risk factor in CVD patients who are already on optimal treatment regimens.^43^ Anti-inflammatory therapies such as canakinumab or colchicine decrease the incidence of CV events in adults with established CVD.^10^^,^^11^ Additional interventions targeting trained immunity or inflammation more broadly might be warranted to address chronic inflammation in children with obesity.^44^

Our study has a number of strengths and limitations. The systems biology approach employed here investigated a number of inflammation related parameters cross-sectionally and longitudinally in the same individual, and has not been previously reported in children. This approach provides complete and novel insights into molecular mechanisms of inflammation and may highlight potential therapeutic targets in children with obesity. We selected participants in an attempt to reduce confounding by, for example, the effects of puberty. On the other hand, this limited our study size too. Future studies should include pre- and peri-pubertal samples with longer follow-up to assess the effects on monocyte activation, innate immune responses, and CVD development.

Limitations include the modest sample size, particularly for the follow-up component, limiting the capacity to assess the impact of weight change over time. Only a single participant obtained optimal weight, leaving us unable to comment on the effects of obesity resolution on inflammatory, immune and cardiometabolic outcomes; these data would be of considerable scientific and clinical interest. We recruited the original cohort from a hospital weight management service, which has specific referral criteria. There is therefore likely to have been some selection bias and this may limit generalizability to the wider population and different race/ethnic groups. Ideally, repeated assessments would provide more detailed trajectory data and long-term follow up would eventually link to hard CVD and metabolic outcomes. Such decades-long longitudinal studies are difficult and resource-intensive and beyond the scope of these unique but preliminary data. Another limitation is the missing information on pubertal status in the control group.

In conclusion, children and adolescents with obesity have a pro-inflammatory phenotype, with heightened systemic inflammation, activated monocytes and increased production of pro-inflammatory cytokines. In contrast, anti-viral responses are sub-optimal in this population. Weight loss does not affect measures of inflammation, and higher inflammation at baseline associated with adverse subclinical CV measures at follow-up. These preliminary data suggest that anti-inflammatory interventions may have a role in reducing the long-term cardiometabolic risks associated with obesity in childhood. Assessment of changes in inflammatory and metabolic measures may therefore be more informative of the effectiveness of interventions than changes in BMI or adiposity.

### Limitations of the study

Limitations include the modest sample size, particularly for the follow-up assessment, limiting the capacity to assess the impact of weight change over time. Only a single participant obtained optimal weight, so the effects of obesity resolution on inflammatory, immune, and cardiometabolic outcomes in children remain unknown; these data would be of considerable scientific and clinical interest. We recruited the original cohort from a hospital weight management service, which has specific referral criteria. This is likely to result in some selection bias, potentially limiting generalizability. Repeated assessments over a longer follow-up period would provide more detailed trajectory data and data on clinical CVD and metabolic outcomes. Data on pubertal status were not available in the control group. It is suggested that overweight or obesity affects the timing of puberty onset,^45^ which could potentially contribute to the differences between groups.

## STAR★Methods

### Key resources table

**Table:** 

REAGENT or RESOURCE	SOURCE	IDENTIFIER
**Antibodies**		

APC-H7-anti human CD14, clone MwP9	BD Biosciences	Cat no. 560270; RRID: AB_1645464
BUV496-anti human CD16, clone 3G8	BD Biosciences	Cat no. 564654; RRID AB_2870224
BUV395-anti human HLA-DR, clone G46-6	BD Biosciences	Cat no. 565972; RRID AB_2738558
BUV805-anti human CD45, clone HI30	BD Biosciences	Cat no. 612892; RRID AB_2744401
PE-Cy7-anti human CD3, clone SK7	BD Biosciences	Cat no. 560910; RRID AB_396896
BV711-anti human CD4, clone SK3	BD Biosciences	Cat no. 563033; RRID AB_2737961
BV480-anti human CD8, clone SK1	BD Biosciences	Cat no. 746470, RRID AB_2743772
BUV737-anti human CD19, clone SJ25C1	BD Biosciences	Cat no. 612757, RRID AB_2716867
BB700-anti human CD56, clone NCAM16.2	BD Biosciences	Cat no. 566574, RRID AB_2744430
BV650-anti human CD69, clone FN50	BD Biosciences	Cat no. 563835, RRID AB_2738442
PE-anti human NKG2C, clone 134591	R&D systems	Cat no. FAB138P-025, RRID AB_2132983
BV786-anti human CCR2, clone LS132.1D9	BD Biosciences	Cat no. 747855, RRID AB_2872317
PE-CF594-anti human CCR5, clone 2D7/CCR5	BD Biosciences	Cat no. 562456, RRID AB_11154599
BB515-anti human CD11b, clone ICRF44	BD Biosciences	Cat no. 564518, RRID AB_2744271
APC-anti human CD11c, clone S-HCL-3	BD Biosciences	Cat no. 340544, RRID AB_400520
BV421-anti human TLR4, clone TF901	BD Biosciences	Cat no. 564401, RRID AB_2738792
BV605-anti human TLR2, clone 11G7	BD Biosciences	Cat no. 742768, RRID AB_2741032

**Chemicals, peptides, and recombinant proteins**		

BD Horizon™ Fixable Viability Stain 700	BD Biosciences	Cat no. #564997
0.5M EDTA	Life Technologies	Cat no. 15575020
human Fc block	BD Biosciences	Cat no. #564220
Fetal Bovine Serum	HyClone (*Invitro* Technologies)	Cat no. SH30071.03
PBS	Oxoid	Cat no. BR0014G
Brilliant Stain buffer	BD Biosciences	Cat no. #566385
Compensation beads	BD Biosciences	Cat no. #552843
Ficoll-Paque	VWR International	Cat no. GEHE17-1440-02
DMSO	Sigma-Aldrich	Cat no. D587-500mL
Nalgene Mr Frosty	Sigma-Aldrich	Cat no. C1562
Roswell Park Memorial Institute (RPMI) 1640	Sigma-Aldrich	Cat no. R0883-500mL
Benzonase Nuclease	Sigma-Aldrich	Cat no. E1014-25KU
Trypan-Blue	Sigma-Aldrich	Cat no. T8154-20mL
Lipopolysaccharide	Sigma-Aldrich	Serotype 055:B5, cat no. L2880
Pam3CSK4	Invivogen	Cat no. tlrl-pms
HEPES	Life Technologies	Gibco, cat no. 15630080
Glutamax	Life Technologies	Cat no. 35050061
Sodium Pyruvate	Life Technologies	Gibco, Cat no. 11360070
Penicillin and streptomycin	Life Technologies	Gibco, cat no. 15140122
Bovine Serum Albumin	Sigma-Aldrich	Cat no. A9576-50ML
TriZol reagent	Invitrogen	Cat no. 15596018

**Critical commercial assays**		

Biorad Bio-Plex Pro Human Cytokine 27-plex Immunoassay kit	Bio-rad	Cat no. M500KCAF0Y
High sensitivity IL-6 ELISA	R&D	Cat no. HS600C
Human C-reactive protein ELISA	R&D	Cat no. DY1707
Pan monocyte isolation kit	MACS Miltenyi	Cat no. 130-096-537
Qiagen RNeasy Micro kit	Qiagen	Cat no. 74004
Illumina TruSeq Stranded mRNA kit	Illumina	Cat no. 20020594
MACS MS cell separation columns	MACS Miltenyi	Cat no. 130-042-201

**Deposited data**		

RNA sequencing data	Database: NCBI Gene Expression Omnibus	GSE226280

**Software and algorithms**		

FlowJo software	BD Biosciences	Version 10.7
Graphpad Prism	Graphpad	Version 9.0
SPSS	SPSS Inc	Version 27.0
R-studio	CRAN	Version 3.5
Bio-Plex Manager	BioRad	N/A
Nanodrop software	Nanodrop	N/A
Bowtie 1	Langmead et al.^46^	http://bowtie-bio.sourceforge.net/index.shtml
MMSEQ	Turro et al.^47^	https://github.com/eturro/mmseq
DEseq2	Love et al.^48^	http://bioconductor.org/packages/elease/bioc/html/DESeq.html
MixOmics	R	Version 1.1.485
Enrichr	Chen et al.^49^	https://maayanlab.cloud/Enrichr/

**Other**		

LSR-II Fortessa X-20	BD Biosciences	N/A
Harpenden stadiometer	Holtain Ltd	N/A
Four-point bio-impedance device	Tanita Corporation	N/A
Glucose analyzer VITROS 5600	Ortho Clinical Diagnostics	N/A
HbA1c analyzer BIO-RAD D10	Biorad	N/A
Sphygmomanometer	Welch Allyn	SN100319095511
SphygmoCor XCEL system	AtCor Medical Pty Ltd	N/A
Roche C8000 system	Roche	Module C702
Biorad TC10 automated cell counter	Biorad	N/A
xPONENT MAGPIX instrument	Biorad	N/A
Bioanalyzer Agilent 2100	Agilent	N/A
DNBBSeq 400 platform	BGI solutions	N/A

### Resource availability

#### Lead contact

Further information and requests for resources and reagents should be directed to David Burgner (David.burgner@mcri.edu.au).

#### Materials availability

This study did not generate new unique reagents.

#### Data and code availability

• Raw data files and count tables for RNAseq are deposited in the NCBI Gene Expression Omnibus under accession numbers GSE226280.
• All original code is available upon request by the lead author. Any additional information required to reanalyze the data reported in this paper is available from the lead contact upon request.
• Any additional information required to reanalyze the data reported in this paper is available from the lead contact upon request.

### Experimental model and study participant details

Participants in the Childhood Overweight Biorepository of Australia (COBRA)^50^ (*n* = 438 cases, hereafter referred to as ‘those with obesity’) were recruited from the Royal Children’s Hospital (Melbourne, Victoria, Australia) Weight Management Service between 2009 and 2018 (*baseline* visit). 98 participants from COBRA consented to ongoing contact and participated in a follow-up study of cardiometabolic phenotypes including a blood sample collection at a mean of 5.8 years later (referred to as COBRA-CardioVascular Risk, COBRA-CVR, *follow-up* visit). Recruitment for COBRA-CVR was from June 2017 through January 2019. Written informed consent was obtained from either the participant or their legally relevant representative. Assent was obtained from participants aged 14 years or older. For the purpose of this study, only postpubertal children were included to limit confounding by pubertal status. Therefore, inclusion criteria for the cases in this study were: i) postpubertal at baseline and <25 years old at follow-up, ii) BMI ≥95^th^ centile at baseline, and iii) viably stored peripheral blood mononuclear cell (PBMC) samples. Exclusion criteria were: i) inability to provide informed consent, ii) immunosuppressive medication within the last 3 months, iii) acute infection requiring medication within 4 weeks of assessment, iv) hsCRP >20 mg/dL (as high hsCRP above 20 is indicative of recent or intercurrent infection).

Control participants (referred to as ‘controls’ hereafter) were healthy control participants from the Pediatric Auto-Immune Disease (PAID) cohort,^51^ who were recruited through the Royal Children’s Hospital (Melbourne, Australia) when attending for elective surgery for non-inflammation-related conditions. Venous blood samples were collected prior to anesthesia. For this study, we selected controls with a BMI *Z* score between −1.5 and 1.5 (defined as healthy weight),^52^ with no inflammatory co-morbidities, matched to the COBRA participants on age, sex, availability of viably stored PBMCs, and sample processing time. The study was approved by the RCH Human Research Ethics Committee, RCH, Melbourne, Australia (HREC Ref. # 28081P and #27127) and is accordance with Helsinki principles.

Clinical data, such as age and sex/gender are reported in table 1. There is no available data on ancestry, race or ethnicity of the study participants. Due to the exploratory nature of this study, no formal sample-size estimation was performed at design of the study.

### Method details

#### Anthropometry measures

Anthropometry was assessed in all cases at baseline and follow-up using the same measurement techniques and devices. Height was measured using a fixed Harpenden stadiometer without shoes. Weight, and percentages of total body fat (%BF) and truncal fat mass (%TF) were measured in light clothes with a four-point bio-impedance device, previously validated for use in children over 6 years.^53^ Body mass index (BMI) was calculated by weight in kg divided by height in meters squared. BMI z-scores and the percentage above the 95^th^ centile (%>95^th^ BMI-centile) based on the US CDC growth reference charts were used.^52^ In brief, the % >95th BMI-centile is the ratio of the individual’s BMI divided by the relevant 95th BMI-centile for an age- and sex-matched individual multiplied by 100%. E.g., the 95th BMI-centile for a male adolescent 14 years is 26 kg/m^2^, such that if this participant’s BMI was 32 kg/m^2^, the % >95th BMI-centile is (32/26) × 100% = 123%.^54^ Waist circumference (WC) was measured with a tape measure at the mid-point between the anterior superior iliac spines and the lower costal border to the nearest 0.5 cm upon expiration. Waist-to-height (WtH) ratio was calculated based on the ratio of waist circumference (in cm) to height (in cm). A pediatric endocrinologist or general pediatrician assessed the Tanner stage for pubertal development, where Tanner 1 was considered pre-pubertal, Tanner 2–3 peri-pubertal, Tanner 4–5 post-pubertal.^55^^,^^56^ In control participants, only height, weight, BMI and BMI z-scores and %>95^th^ centile were recorded.

#### Cardiovascular risk factors

For COBRA-CVR, glucose and HbA1c were analyzed using standard methods (in fluoride oxalate plasma on VITROS 5600 and EDTA plasma on BIO-RAD D10, respectively). Type 2 diabetes mellitus or prediabetes was defined according to the American Diabetes Association criteria^57^; no participant had diabetes.

#### Blood pressure and heart rate

Blood pressure measurements at visit 1 were performed with a manual sphygmomanometer by auscultation of the right brachial artery when the individual was sitting, quiet and calm. At visit 2 (COBRA-CVR), right brachial systolic blood pressure (SBP), diastolic (DBP), mean arterial pressure (MAP), and pulse pressure (PP) and heart rate (HR) was measured using a SphygmoCor XCEL system after a 5 min rest in supine position. The mean of 3 measurements was used. The cuff-size were fitted according to published clinical practice guidelines and the updated 2017 clinical guidelines were used to define normal or elevated blood pressure and stage I and II hypertension.^58^

#### Subclinical cardiovascular measures

Subclinical cardiovascular measures in COBRA-CVR participants at follow-up included the non-invasive assessment of carotid intima-media thickness (cIMT), carotid-femoral pulse wave velocity (PWV), and carotid elasticity. These measures are considered intermediate or surrogate outcomes on the pathway and predictive for future cardiovascular disease endpoints. On-site standardized protocols were used for the assessment of subclinical cardiovascular measures as previously described in detail.^59^ In brief, cIMT was assessed on the right carotid artery in the supine participant according to on-site standardized protocols^60^ with simultaneous electrocardiogram (ECG) gating to assess cIMT at end-diastole (R-wave of ECG^61^). PWV was determined by the SphygmoCor XCEL device in the supine participant after a 5-min rest. The mean out of 3 measures was used for analysis.^60^ Carotid elasticity was calculated as the percentage change in lumen diameter per deltaP of 10 mmHg (i.e., %/10 mmHg). The maximum and minimum lumen diameter was calculated using Carotid Analyzer (Medical Imaging Applications, Coralville, Iowa, USA), a semiautomatic edge-detection software program. DeltaP was calculated as the difference between systolic and diastolic blood pressure.^59^^,^^60^

#### Biobanking procedures and analysis of plasma

For cases and controls, peripheral blood samples were collected into EDTA, kept at room temperature and processed at the biobanking facility at Murdoch Children’s Research Institute within 4 h. Blood vacutainers were centrifuged for 10 min at 3800 rpm, room temperature. Plasma was removed within 2 h and stored at −80°C until analysis. PBMCs were isolated by differential centrifugation using a standard Ficoll procedure. Subsequently, PBMCs were resuspended in 90% Heat Inactivated (HI) Fetal Bovine Serum (FBS) + 10% DMSO, chilled slowly to −80°C using a Mr Frosty and then transferred to vapour-phase liquid nitrogen storage to maintain cell viability.

For COBRA and COBRA-CVR participants, white blood cell counts were performed immediately upon blood withdrawal using automated cell counters. Plasma lipid profiles were measured for all participants in stored plasma using the Roche C8000 system (module C702): Total cholesterol and triglycerides were assessed with enzymatic reactions (cholesterol esterase and oxidase), HDL cholesterol was assessed using accelerator selective detergent (HDL-cholesterol Gen.4 test) and LDL cholesterol was calculated with the Friedewald formula.^62^

#### PBMC thawing

PBMCs were thawed and resuspended in Roswell Park Memorial Institute 1640 (RPMI) supplemented with 10% HI-FBS and 25 U/mL Benzonase at 37°C. Cells were washed once in RPMI+10%HI-FBS, resuspended in RPMI and counted using a Biorad TC10 automated cell counter, using a ½ dilution in Trypan-Blue (0.4% in 0.81% sodium chloride and 0.06% potassium phosphate dibasic solution, sterile filtered) to distinguish viable cells. Mean viability of PBMCs was 71% and was not associated with the age of the sample when comparing obese (mean age of sample 7.3 ± 1.9 years) vs. controls (mean age of sample 9.2 ± 1.1 years, Figure S1A). Viability in follow-up samples (COBRA-CVR) was 78% with mean age of the samples 1.4 ± 0.5 years.

#### Stimuli and reagents

Stimuli in PBMC cultures included lipopolysaccharide (LPS) and Pam3Cys. Cells were cultured in RPMI supplemented with 20 mM HEPES, Glutamax, 1 mM Sodium Pyruvate, 50uM uridine and penicillin and streptomycin (complete RPMI).

#### PBMC stimulation assays

2.5 × 10^5^ PBMCs/well were stimulated in round-bottom 96-well plates with either complete RPMI as negative control, 10 ng/mL LPS (TLR4) or 10 μg/mL Pam3Cys (TLR2) at 37C, 5%CO2 to study *ex vivo* cytokine production capacity. After 24 h, plates were centrifuged for 8 min at 350 g and 80 μl supernatant was removed and stored at −80C until cytokine measurement.

#### Cytokine measurements

Cytokines were quantified using the Bio-Rad Bio-Plex Pro Human Cytokine 27-plex Immunoassay kit and detection software, with small modifications to manufacturer instructions. Standards were prepared in a dilution series using the standard kit-provided diluent. Supernatants were thawed, diluted 4 times and added to plates that contained 8 standards in duplicate (including blank), and inter-plate controls. Supernatants from each participants were measured on the same plate but participants were randomly distributed over plates to prevent batch effects. Plates were analyzed using the xPONENT MAGPIX instrument. Standard curves were produced based on the standard dilutions and optimised for a recovery rate of 70–130%. Final measures in pg/mL were derived by the Bio-Plex Manager Software using the standard curves. Cytokine concentrations above the upper limit of quantification (ULOQ) of the standard curve were excluded whereas cytokine concentrations below the lower level of quantification (LLOQ) were replaced with a value equal to 50% of LLOQ.

Plasma IL-6 (pg/mL) was measured using high sensitivity ELISA and plasma hsCRP (mg/dL) was determined using the Human C-Reactive Protein ELISA according to the manufacturer’s instructions.

#### Monocyte isolation and stimulation

Monocytes were isolated from PBMCs using negative selection with the Pan monocyte isolation kit and MACS cell separation columns according to the manufacturer’s instructions. In short, PBMCs were centrifuged and resuspended in cold MACS buffer (0.5% BSA in PBS, supplemented with 2 mM EDTA). Fc-receptor blocking agent and antibodies were added and incubated for 5 min. Anti-Biotin microbeads were subsequently added and incubated for 10 min. After column separation, the flow through was collected and centrifuged. Monocytes were resuspended in RPMI and counted using the Bio-Rad TC10 automated cell counter. Trypan-Blue was used to assess cell viability. Mean viability of the monocytes was 86% (91% at follow-up) and purity was >95% as measured by flow cytometry (data not shown). 200.000 cells were immediately centrifuged and resuspended in 500ul TriZoL for RNA isolation. When cell counts allowed, a further 250.000 monocytes were either stimulated with RPMI or 10 ng/mL LPS for 4 h, after which the cells were centrifuged and resuspended in TriZOL for RNA isolation.

#### RNA isolation and sequencing

Total RNA purification was performed using the Qiagen Rneasy Micro kit according to the manufacturer’s instructions. Prior to on-column isolation, RNA was phase extracted from TriZOL using chloroform extraction. In short, 100 μl of chloroform was added to 500 μl TriZOL. Samples were vigorously shaken for 15–30 s and left on the countertop for 5 min to separate the layers. Samples were centrifuged for 15 min at 4C, 12000 g and the top layer (containing the RNA) was transferred to a new Eppendorf. Equal volumes of 70% ethanol were added and samples were transferred to the Rneasy Micro isolation columns for further purification. On-column DNAse I treatment was performed. RNA concentrations were first measured using NanoDrop software and further quality control was performed using the Bioanalyzer Agilent 2100. Isolated RNA was sent for next-generation sequencing on the DNBSeq 400 platform. Libraries were prepared using the Illumina TruSeq Stranded mRNA kit with a starting input of 100 ng (where available) and sequenced, with the generation of approximately 20 million 100-bp paired-end reads per sample. Raw data files and count tables for RNAseq are deposited in the NCBI Gene Expression Omnibus under accession numbers GSE226280.

#### RNA sequencing analysis

Raw data were received as fastq files and quality checked using FastQC. Reads were aligned to the human transcriptome hg19 using Bowtie^46^ and gene expression quantification was performed using mmSeq.^47^ DESeq2^48^ was used to normalize counts per gene and Reads Per Kilo base per Million mapped reads (RPKM) values were used for plotting gene expressions in heatmaps and graphs. DESeq2 was further used to assess differentially expressed genes (DEGs) using logistic regression with cut-offs of RPKM>5, fold change>2 and adjusted *p*-value<0.05 as significant. Principal Component Analaysis (PCA), t-distributed stochastic neighbor embedding (tSNE) and Volcano plots visualization was performed using R-studio version 1.1.485 package MixOmics. Pairwise comparisons were made between 4 h medium stimulated vs. 4 h LPS stimulated and unpaired grouped comparisons between obese vs. controls. Gene ontology and pathway analysis of DEGs were performed using Enrichr.^49^

#### Flow cytometry

300 000 PBMCs were resuspended in 100 μL PBS and stained with BD Horizon Fixable Viability Stain 700 (1/1000) for 15 min at RT, protected from light. Cells were washed by adding 5 mL of FACS buffer (2% FBS in PBS+2 mM EDTA), centrifuged and subsequently resuspended in 50 μl FACS buffer containing human Fc block. After 5–10 min incubation, 50 μl antibody mix (1/50 anti-CD14, 1/50 CD16, 1/50 anti-HLA-DR, 1/50 anti-CD45, 1/50 anti-CD3, 1/50 anti-CD4, 1/25 anti-CD8, 1/50 anti-CD19, 1/50 anti-CD56, 1/50 anti-CD69, 1/25 anti-NKG2C, 1/50 anti-CCR2, 1/50 anti-CCR5, 1/50 anti-CD11b, 1/100 anti-CD11c, 1/100 anti-TLR4, 1/25 anti-TLR2 in FACS buffer supplemented with Brilliant Stain buffer) was added and incubated for 30 min at room temperature in the dark. Cells were washed with 1 mL FACS buffer, centrifuged and resuspended in 350 μL FACS buffer. Samples were measured on an LSR-II Fortessa X-20 and analyzed using FlowJo software. Compensation was performed at the time of sample acquisition using compensation beads. Median fluorescence intensity was obtained using Flow-minus-one (FMO) controls. The FCS files underwent pre-processing to remove debris, doublets, dead cells and non-hematopoeietic cells (based on CD45 expression). Processed files were then analyzed by manual gating and unsupervised computational methods in parallel using FlowJo software. For unsupervised computation analysis, the preprocessed FCS files were randomly downsampled to 20,000 events and subsequently concatenated to a single file containing all events. The groups were labeled to be able to separate them after analysis. Unsupervised clustering was performed based on the relevant values of 16 markers (CD3, CD4, CD8, CD19, CD14, CD16, CD56, HLA-DR, CD69, CCR2, CCR5, NKG2C, CD11b, CD11c, TLR4 and TLR2) using the FlowSOM plugin in FlowJo. A pre-specified number of 20 clusters were used in the meta-clustering. The marker expression levels per cluster were visualized in a heatmap after normalization of each marker between 0 and 1 using the *caret* package in R. Based on expression patterns, clusters were manually annotated. To visualize the high dimensional data, tSNE analysis was performed using the tSNE plugin in FlowJo with parameters set on 1000 iterations and a perplexity of 30. FlowSOM clusters were then layered on top of the tSNE to visualize the different clusters in the created tSNE. The contribution of the different study groups (those with obesity or controls) to the total tSNE was then inspected by separating the tSNE in different figures, one for each group. Visual differences were confirmed and statistically analyzed by manual gating, also using the clusters defined by FlowSOM (gating strategy see Figure S1B). Characterization of monocytes subsets based on CD14 and CD16 expression is according to current recommendations.^63^^,^^64^

### Quantification and statistical analysis

Data were expressed as mean (standard deviation), median (range) or number (percentage), unless stated otherwise in the figure legends. Normality checks and tests for heteroscedasticity were performed by inspecting qq-plots and statistical tests using graphpad. Differences in clinical characteristics and monocyte phenotype and function between those with obesity and controls were assessed with Student’s t-tests or Mann–Whitney U tests depending on normality. The *p*-values were adjusted for multiple comparisons using the Benjamini and Hochberg approach to control the false discovery rate (FDR). FDR-adjusted *p* < 0.05 were considered significant. Differences in measures between visit 1 and visit 2 were analyzed using paired statistics; a paired t-test or Wilcoxin signed rank tests depending on normality. The effects of sex on all outcomes was assessed using ANOVA.

In COBRA-(CVR) partipants, the association between change in BMIz between time points and change in inflammation measures was investigated with linear regression models adjusted for sex and age at each time point. Linear regression models adjusted for sex, age, and BMIz at the relevant time point(s) were used to investigate the associations of (i) baseline inflammation measures and (ii) follow-up inflammation measures with follow-up subclinical cardiovascular measures. Exposure and outcome variables were scaled to a standard distribution (standard deviation units) for visualization on forest plots. In sensitivity analyses, one participant at follow-up was excluded.

Data were analyzed using Prism version 9.0, SPSS version 27.0 and R-studio version 3.5.

## Supplemental information

Document S1. Figures S1 and S2 and Tables S1–S4

Table S5. Differentially expressed genes comparing controls (unstimulated and LPS stimulated) with obese (unstimulated and LPS stimulated) RPMI>5 and padj<0.05 as cut-offs, related to Figure 3.

Table S6. GO pathways and associated genes in unstimulated cells, obese vs. controls Upper panel: downregulated in obese/upregulated in controls. Lower panel: upregulated in obese / downregulated in controls, related to Figure 3 and Table S5 and S7.

Table S7. GO pathways and associated genes in LPS stimulated cells, obese vs. controls Upper panel: downregulated in obese/upregulated in controls. Lower panel: upregulated in obese / downregulated in controls, related to Figure 3 and Table S5 and S6.

Table S8. Associations of change of BMIz over time (exposure) vs. inflammatory measures (outcome) Unadjusted p-values are reported, related to Figure 4.

Table S9. Associations of baseline inflammatory measures (exposure) with subclinical cardiovascular measures (outcome) Unadjusted p-values are reported, related to Figure 4.
