# Supplementary material for: Increased innate immune responses in adolescents with obesity and its relation to subclinical cardiovascular measures: An exploratory study
Source: iScience. 2024 Apr 23;27(5):109762. doi: 10.1016/j.isci.2024.109762 (PMC11089376; doi:10.1016/j.isci.2024.109762)
Supplement: Document S1. Figures S1 and S2 and Tables S1–S4 [file mmc1.pdf]

iScience, Volume *27*

Supplemental information

Increased innate immune responses in adolescents with obesity and its relation to subclinical cardiovascular measures: An exploratory study

Siroon Bekkering, Christoph Saner, Boris Novakovic, Toby Mansell, Danielle K. Longmore, Zoe McCallum, Anne-Louise Ponsonby, Markus Juonala, Mihai G. Netea, Matthew A. Sabin, Richard Saffery, Niels P. Riksen, and David P. Burgner

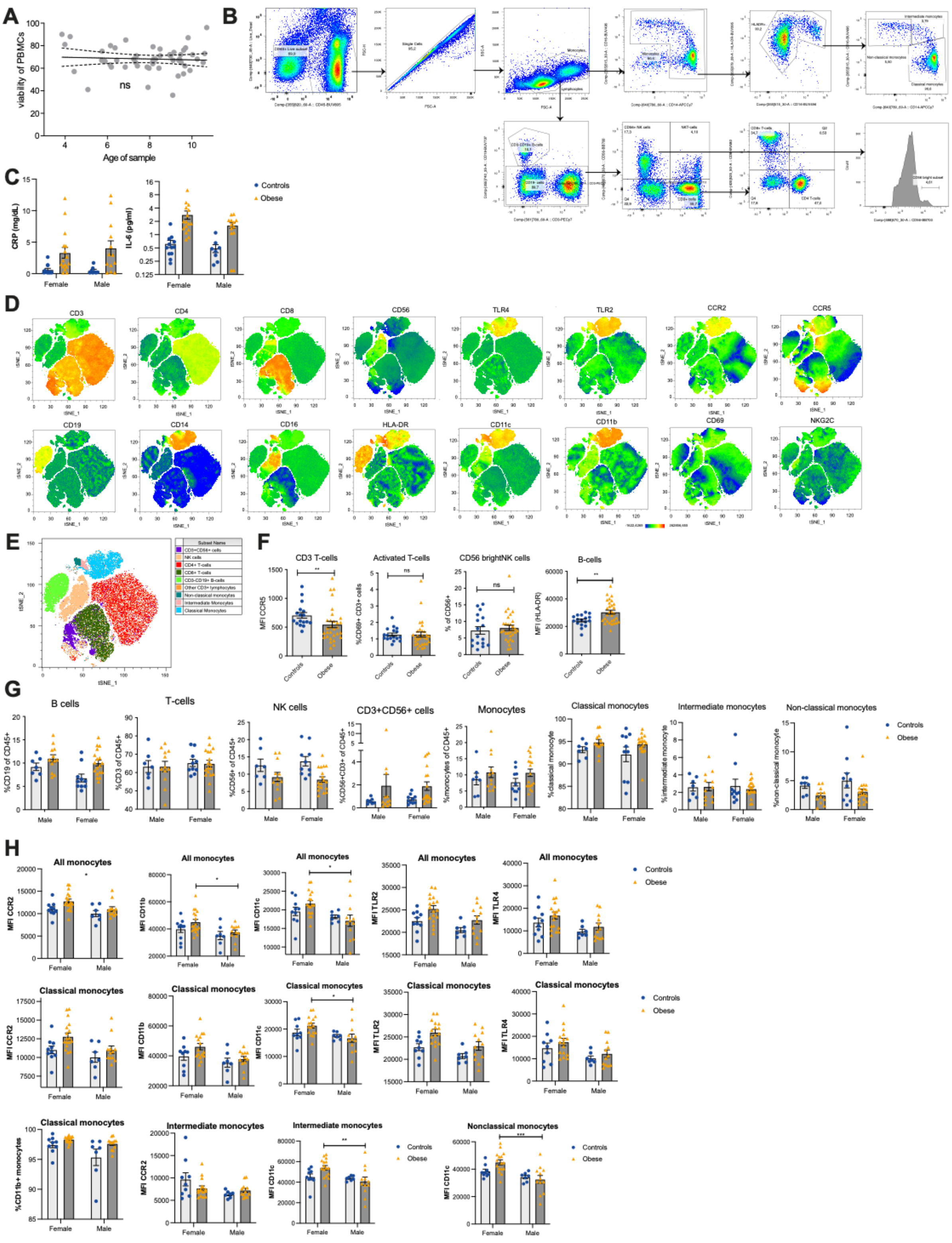

Supplementary Figure 1. Related to figure 1. A. Viability of PBMCs vs age of sample. B. Gating strategy for manual gating. C. Sex differences in plasma inflammation. D. tSNE plots per flow cytometry marker. E. Manual gating confirmed cellular subtypes in tSNE. F. Activation markers in T cells, NK cells and B cells. G. Sex differences in cellular subtypes as measured by flow cytometry. H. Sex differences in monocyte activation markers as measured by flow cytometry. Those with obesity are indicated in yellow and controls in blue. Figures show all individual data plus bars showing the mean and Standard Error of the Mean (SEM).

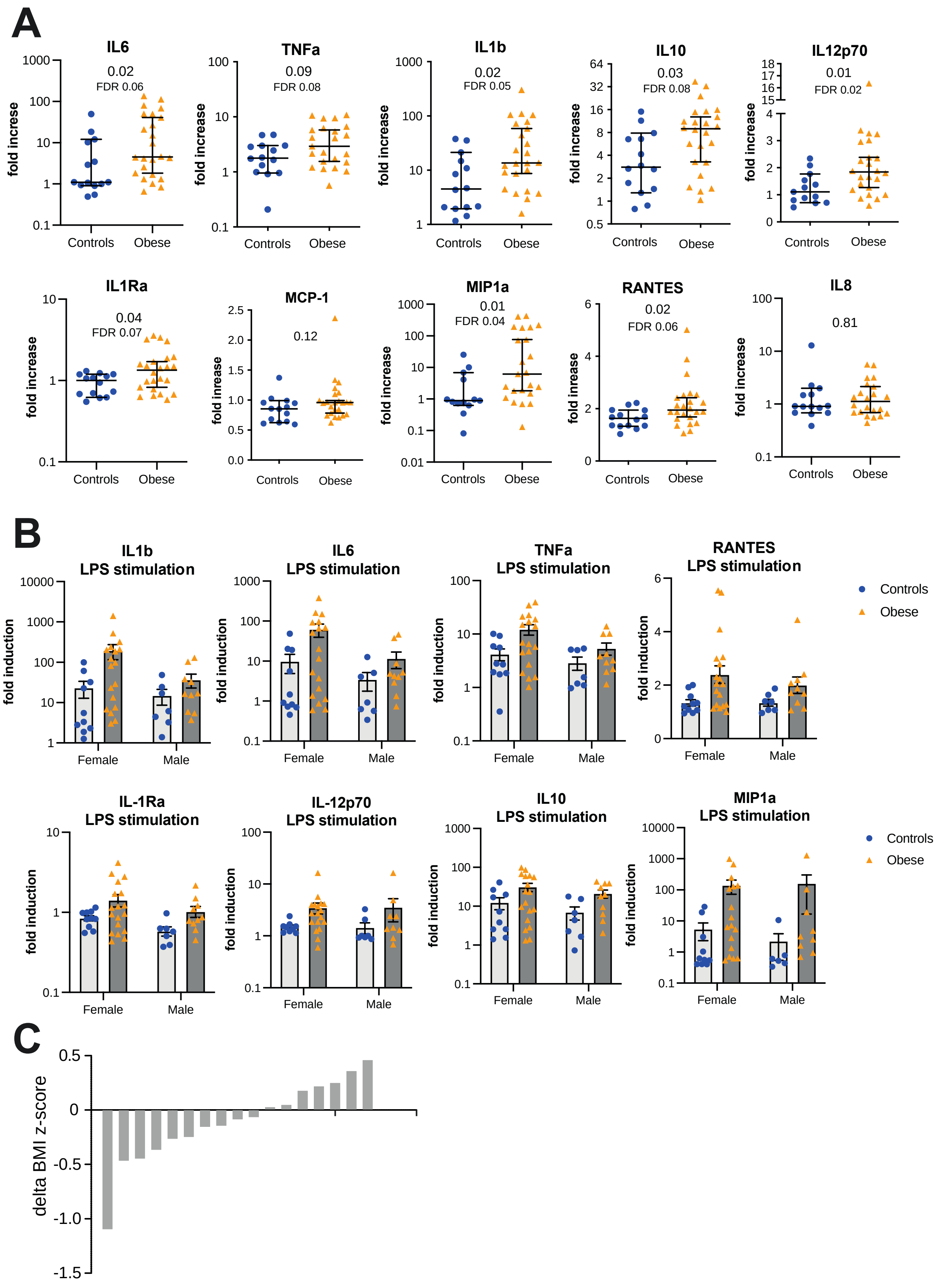

Supplementary Figure 2. Related to figure 2. A. Individual values of innate cytokine production capacity for Pam3Cys stimulated PBMCs. Children with obesity are indicated in yellow and controls in blue. Figures show all individual data plus bars showing the mean and Standard Error of the Mean (SEM). B. Sex differences for

innate cytokine production capacity upon LPS stimulation. C. Change in BMIz score for each individual participant.

Table S1 - Monocyte activation markers

Monocyte Subset	Variable	P-val	Adjusted P-val
All_monocytes	CCR2_perc	0,0001	0,00192308
Intermediate	CCR5_MFI	0,0004	0,00384615
Classical	CCR2_perc	0,0006	0,00576923
Intermediate	HLADR_MFI	0,0006	0,00769231
All_monocytes	TLR2_Perc	0,0008	0,00961538
Intermediate	CD11b_MFI	0,0008	0,01153846
Intermediate	TLR2_MFI	0,0008	0,01346154
All_monocytes	CD11b_perc	0,0012	0,01538462
Nonclassical	CD11b_perc	0,0036	0,01730769
Nonclassical	TLR2_MFI	0,0037	0,01923077
Intermediate	CD11b_perc	0,0059	0,02115385
Intermediate	CCR2_perc	0,0065	0,02307692
All_monocytes	TLR2_MFI	0,0068	0,025
Intermediate	TLR4_MFI	0,0069	0,02692308
Classical	TLR2_Perc	0,0073	0,02884615
Intermediate	TLR2_Perc	0,0081	0,03076923
Nonclassical	HLADR_MFI	0,0084	0,03269231
Classical	TLR2_MFI	0,0094	0,03461538
Classical	CCR2_MFI	0,0176	0,03653846
All_monocytes	CCR2_MFI	0,0179	0,03846154
Nonclassical	TLR2_Perc	0,0232	0,04038462
Intermediate	CCR5_perc	0,0277	0,04230769
Classical	CD11b_perc	0,0297	0,04423077
Nonclassical	CD11b_MFI	0,0426	0,04615385
All_monocytes	CCR5_MFI	0,0663	ns
Intermediate	CD11c_perc	0,0863	ns
Classical	CCR5_MFI	0,0890	ns
Classical	CD11b_MFI	0,0916	ns
All_monocytes	CD11b_MFI	0,0932	ns
Intermediate	TLR4_perc	0,1007	ns
Nonclassical	TLR4_MFI	0,1060	ns
All_monocytes	HLADR_MFI	0,1185	ns
Intermediate	CD11c_MFI	0,1439	ns
All_monocytes	TLR4_MFI	0,1441	ns
Classical	HLADR_MFI	0,1613	ns
Classical	TLR4_MFI	0,1700	ns
Nonclassical	CD11c_MFI	0,1946	ns
Nonclassical	TLR4_perc	0,3355	ns

Classical	CD11c_MFI	0,3783	ns
All_monocytes	TLR4_perc	0,3988	ns
Classical	CCR5_perc	0,4001	ns
Nonclassical	CCR5_perc	0,4876	ns
All_monocytes	CD11c_MFI	0,4895	ns
All_monocytes	CCR5_perc	0,5280	ns
All_monocytes	CD11c_perc	0,5583	ns
Classical	CD11c_perc	0,5990	ns
Intermediate	CCR2_MFI	0,6028	ns
Classical	TLR4_perc	0,6518	ns
Nonclassical	CCR2_MFI	0,6910	ns
Nonclassical	CCR5_MFI	0,8606	ns
Nonclassical	CCR2_perc	0,9500	ns
Nonclassical	CD11c_perc	0,9823	ns

Table S1. Monocyte activation markers (including FDR adjusted p-values). Related to Figure 1.

Table S2 - Sex differences in monocyte activation markers

**Measure**	**Group pval**	**Sex pval**	**Interaction pval**
B-cells (% of CD45)	**0,004**	**0,04**	0,38
T-cells (% of CD45)	0,94	0,48	0,94
NK-cells (% of CD45)	**0,001**	0,86	0,46
CD3+CD56+-cells (% of CD45)	**0,048**	0,97	0,90
Monocytes (% of CD45)	0,10	0,71	0,79
Classical monocytes (% of total monocytes)	**0,047**	0,44	0,76
Intermediate monocytes (% of total monocytes)	0,75	0,91	0,68
Nonclassical monocytes (% of total monocytes)	**0,02**	0,31	0,88
**Total Monocytes**	**Group pval**	**Sex pval**	**Interaction**
CCR2%	**<0,0001**	0,20	0,55
CCR2 MFI	**0,02**	**0,02**	0,51
CCR5%	0,95	0,24	0,57
CCR5 MFI	**0,03**	0,81	**0,06**
HLADR MFI	0,33	0,78	0,16
CD11b%	**0,0005**	0,29	0,80
CD11b MFI	0,10	**0,01**	0,54
CD11c%	0,55	0,13	0,25
CD11c MFI	0,64	**0,01**	0,18
TLR2%	**0,003**	0,57	0,96
TLR2 MFI	**0,006**	**0,01**	0,80
TLR4%	0,21	0,81	0,54
TLR4 MFI	0,15	**0,02**	0,79
**Classical Monocytes**	**Group pval**	**Sex pval**	**Interaction**
CCR2%	**0,0002**	0,08	0,38
CCR2 MFI	**0,02**	**0,02**	0,50
CCR5%	0,87	0,45	0,84
CCR5 MFI	**0,02**	0,66	0,12
HLADR MFI	0,34	0,86	0,19
CD11b%	**0,004**	**0,01**	0,19
CD11b MFI	0,08	**0,01**	0,42
CD11c%	0,52	0,18	0,39
CD11c MFI	0,49	**0,02**	0,18
TLR2%	**0,04**	0,31	0,78
TLR2 MFI	**0,003**	**0,006**	0,55
TLR4%	0,73	0,34	0,72
TLR4 MFI	0,21	**0,01**	0,86

**Intermediate Monocytes**	**Group pval**	**Sex pval**	**Interaction**
CCR2%	**0,02**	0,37	0,70
CCR2 MFI	0,49	**0,02**	0,07
CCR5%	**0,03**	0,94	0,89
CCR5 MFI	**0,02**	0,68	0,91
HLADR MFI	**0,001**	0,93	0,97
CD11b%	**0,005**	0,10	0,75
CD11b MFI	**0,002**	0,12	0,61
CD11c%	0,05	0,34	0,40
CD11c MFI	0,34	**0,02**	0,07
TLR2%	**0,02**	0,24	0,57
TLR2 MFI	**0,001**	0,14	0,17
TLR4%	0,46	0,09	0,78
TLR4 MFI	**0,03**	0,20	0,60
**Non classical Monocytes**	**Group pval**	**Sex pval**	**Interaction**
CCR2%	NA	NA	NA
CCR2 MFI	NA	NA	NA
CCR5%	0,12	0,39	0,20
CCR5 MFI	0,65	0,72	0,64
HLADR MFI	**0,01**	0,72	0,45
CD11b%	0,003	0,30	0,50
CD11b MFI	**0,048**	0,81	0,72
CD11c%	0,31	0,25	0,08
CD11c MFI	0,31	**0,0009**	0,08
TLR2%	0,06	0,21	0,75
TLR2 MFI	**0,008**	0,49	0,41
TLR4%	0,46	0,21	0,62
TLR4 MFI	0,09	0,30	0,54

Table S2. Sex differences in monocyte activation markers as measured by flow cytometry. Related to Figure 1

Table S3 - Cytokine production capacity

		P-val	Adj p-val
Fold_LPS	MIP1a/CCL3	0,0005	0,005
Fold_LPS	RANTES	0,0021	0,01
Fold_LPS	IL-1b	0,0041	0,015
Fold_Pam3Cys	IL-12	0,0114	0,02
Fold_LPS	IL-12	0,0133	0,025
Fold_LPS	IL-6	0,0144	0,03
Fold_Pam3Cys	MIP1a/CCL3	0,0162	0,035
Fold_LPS	IL-10	0,0164	0,04
Fold_Pam3Cys	IL-1b	0,0175	0,045
Fold_LPS	MCP-1	0,0186	0,05
Fold_Pam3Cys	RANTES	0,0206	ns
Fold_LPS	TNFa	0,0211	ns
Fold_Pam3Cys	IL-6	0,0223	ns
Fold_Pam3Cys	IL-1Ra	0,0261	ns
Fold_Pam3Cys	IL-10	0,0282	ns
Fold_LPS	IL-1Ra	0,0383	ns
Fold_Pam3Cys	TNFa	0,088	ns
Fold_Pam3Cys	MCP-1	0,1191	ns
Fold_LPS	IL-8	0,3551	ns
Fold_Pam3Cys	IL-8	0,81	ns

Table S3. Cytokine production capacity (including FDR adjusted p-values). Related to Figure 2

Table S4 - Sex differences in cytokine production capacity

Measure	Group pval	Sex pval	Interaction
IL-10 LPS	0,0274	0,2865	0,7376
IL-1b LPS	0,1682	0,2349	0,2843
IL-6 LPS	0,1314	0,1561	0,269
TNFa LPS	0,0419	0,1104	0,2757
MIP1a LPS	0,1065	0,9272	0,8978
GCSF LPS	0,1201	0,2126	0,2864
RANTES LPS	0,0122	0,5459	0,5469
IL-1Ra LPS	0,0325	0,1612	0,7769
IL12p70 LPS	0,0603	0,9954	0,9609
MCP1 LPS	0,0101	0,9929	0,4804
IL-10 P3C	0,0635	0,2785	0,7808
IL-1b P3C	0,165	0,2563	0,2418
IL-6 P3C	0,1594	0,0888	0,2578
TNFa P3C	0,071	0,13	0,341
MIP1a P3C	0,178	0,168	0,195
GCSF P3C	0,179	0,247	0,298
RANTES P3C	0,062	0,664	0,581
IL-1Ra P3C	0,056	0,134	0,74
IL12p70 P3C	0,113	0,707	0,44
MCP1 P3C	0,189	0,362	0,929

Table S4. Sex differences in cytokine production capacity. Related to Figure 2
